# Development and Validation of Spectrophotometric Method for Determination of Levofloxacin in Rat Plasma

**DOI:** 10.3390/molecules31050869

**Published:** 2026-03-05

**Authors:** Tahir Suleymanov, Emilya Balayeva, Elnur Gasimov, Aytac Badalova, Kubra Aliyeva

**Affiliations:** 1 Department of Pharmaceutical Chemistry, Azerbaijan Medical University, Baku 1022, Azerbaijan; ebalayeva@amu.edu.az; 2 Department of General Surgery, Azerbaijan Medical University, Baku 1022, Azerbaijan; elnurgasimov@rambler.ru; 3 Department of Pathophysiology, Azerbaijan Medical University, Baku 1022, Azerbaijan; aytactahir001@gmail.com; 4 Department of Pharmaceutical Technology and Management, Azerbaijan Medical University, Baku 1022, Azerbaijan; kubra.aliyeva@mail.ru

**Keywords:** validation, levofloxacin prolonged, blood, ultraviolet–visible spectrophotometry, linearity, accuracy, hernia mesh

## Abstract

A simple, rapid, and cost-effective UV–Vis spectrophotometric method was developed and validated for the determination of levofloxacin in rat plasma to support the evaluation of a novel antimicrobial mesh implant containing levofloxacin, chitosan, gelatin, tinctura propolis, citric, acid and glycerin. Plasma samples were treated with 0.1 M HCl, and absorbance was measured at 290 nm. The method was validated according to FDA and ICH guidelines, including assessments of linearity, sensitivity, accuracy, precision, and specificity. The calibration curve was linear over the concentration range of 2.5–12.5 μg/mL (R^2^ = 0.999, *p* < 0.001). The limit of detection and limit of quantification were 0.21 μg/mL and 0.62 μg/mL, respectively. Intra- and inter-day precision showed low relative standard deviation values (0.2% and 0.25%), while recovery ranged from 94.8% to 96.4%, confirming acceptable accuracy. No significant interference from plasma matrix components was observed. Compared with chromatographic techniques, the proposed method provides an accessible alternative for routine bioanalysis and therapeutic monitoring. The validated assay is suitable for assessing prolonged levofloxacin release from implantable drug delivery systems in preclinical studies.

## 1. Introduction

The development of new analytical methods for the determination of medicinal substances in biological fluids is a significant aspect in pharmaceutical analysis. The accurate quantification of active pharmaceutical ingredients requires the development and validation of reliable analytical methods. These methods are fundamental for ensuring the quality, safety, and therapeutic efficacy of pharmaceutical products [1,2,3,4]. The reliable quantification of antimicrobial agents in biological matrices is essential in pharmaceutical research, therapeutic monitoring, and the evaluation of novel drug delivery systems. Accurate measurements of antibiotic levels in plasma provide crucial information on pharmacokinetic behavior, systemic exposure, and safety, which contributes to optimal therapeutic dosing and helps prevent antimicrobial resistance [5]. This need is particularly pronounced in studies involving sustained or controlled release systems, where drug concentrations in vivo must be reliably monitored. Levofloxacin is a widely used fluoroquinolone antibiotic characterized by broad spectrum activity against Gram-positive and Gram-negative bacteria. It acts by inhibiting bacterial DNA gyrase and topoisomerase IV, thereby preventing bacterial DNA replication and transcription. Due to its concentration-dependent bactericidal activity and favorable pharmacokinetic properties, levofloxacin is widely prescribed for respiratory tract, urinary, and soft tissue infections [6]. Precise quantification of levofloxacin in plasma is especially important when evaluating new drug delivery systems that aim to sustain therapeutic concentrations at the target site over extended periods. In surgical practice, mesh implants are commonly employed in hernia repair procedures to reinforce weakened tissues. However, postoperative infections remain a significant complication. To address this clinical challenge, research has focused on incorporating antimicrobial agents directly into implant materials to achieve localized and prolonged antimicrobial action at the surgical site. A novel composite mesh system containing levofloxacin entrapped within a biopolymeric matrix composed of chitosan, pectin, and glycerin has been developed to enhance antimicrobial protection. The preclinical evaluation of such a system requires a dependable analytical method capable of detecting and quantifying levofloxacin in plasma samples following implantation. Several analytical techniques have been described for the determination of levofloxacin, including high-performance liquid chromatography (HPLC), electrochemical detection, fluorescence methods, and spectrophotometric measurements. Chromatographic methods are highly sensitive and selective but often involve expensive instrumentation, complex sample preparation, and specialized technical expertise [7]. These practical limitations can restrict their routine use, especially in laboratories with constrained resources. In contrast, UV–Vis spectrophotometry provides a practical analytical alternative due to its simplicity, rapid analysis time, and relatively low operational cost. When properly optimized and validated, spectrophotometric methods can provide reliable quantitative results suitable for routine bioanalytical work. Although spectrophotometric techniques have been applied for the determination of levofloxacin in pharmaceutical formulations, fewer studies have addressed its measurement in biological matrices using simple, reproducible spectrophotometric procedures. In addition, analytical protocols specifically designed to evaluate prolonged drug release from implantable systems remain limited. Therefore, there is a clear need for a validated, efficient, and cost-effective method for plasma analysis in preclinical investigations. The objective of the present study was to develop and validate a UV–Vis spectrophotometric method for the determination of levofloxacin in rat plasma. The method was optimized with respect to solvent selection, wavelength detection, and sample preparation procedures, and subsequently validated in accordance with internationally recognized regulatory guidelines [8,9]. The proposed analytical approach is intended to support the pharmacokinetic assessment and monitoring of sustained levofloxacin release from the novel antimicrobial mesh implant, providing a reliable analytical tool for preclinical and pharmaceutical research.

## 2. Materials and Methods

An experimental laboratory study was performed to evaluate the applicability of the validated spectrophotometric method to rat plasma samples. Our aim was to spectrophotometrically determine the amount of levofloxacin over time in blood administered as part of a hernia mesh. “This study was reviewed and approved by the Ethical Committee Board of Azerbaijan Medical University (Protocol No. 28; 5 July 2023) and was conducted in accordance with the principles of the Declaration of Helsinki.”

### 2.1. Surgical Implantation of Levofloxacin-Loaded PVDF Mesh in Rats and Experimental Protocol

The rats were anesthetized with calypsol, and a ~3 cm midline incision was made through the abdominal skin and subcutaneous tissue under non-sterile conditions.

The aponeurosis of the rectus muscles was bluntly separated from the surrounding tissues, and a 1.5 × 1.0 cm mesh implant (PVDF—polyvinylidene fluoride) impregnated with a biopolymer–antibiotic complex (levofloxacin) was placed in an overlay position over the aponeurosis and fixed at four points using atraumatic sutures. The skin was then closed. To evaluate the prolonged activity and blood concentration of levofloxacin, blood samples were collected on days 1, 3, 5, 7, and 10 of the experiment and analyzed using a UV–visible spectrophotometric method. For this purpose, the rats were euthanized according to generally accepted guidelines by the administration of a high dose of calypsol. Approximately 2.0 mL of blood was collected from each animal into special test tubes containing 3.2% sodium citrate.

### 2.2. Materials and Apparatuses

The levofloxacin standard was provided by KRKA, d. d., Novo Mesto, SI (serial number: UG1456). Hydrochloric acid was obtained from Merck, Darmstadt, DE (serial number: I674930 308). The hernia mesh implant containing levofloxacin, chitosan, gelatin, tinctura propolis, citric acid and glycerin was developed at Azerbaijan Medical University (Baku, Azerbaijan). Blood samples for analysis were provided by laboratory of Teaching Surgery Clinic of Azerbaijan Medical University. UV spectra were obtained by UV–Visible double-beam spectrophotometer (Agilent Technologies Cary 60-2, Santa Clara, CA, USA) with 1 cm matched quartz cells. The absorption spectra of reference and test solution were obtained in a 1 cm quartz cell over a range of 200–400 nm.

### 2.3. Solubility Test of Levofloxacin

The solubility of the levofloxacin sample (50 mg) was investigated in double-distilled water, methanol, ethanol, 1 N NaOH, acetonitrile and 0.1 N HCl. The drug was found to be soluble in 0.1 N HCl.

The standard stock and working solutions of levofloxacin were prepared using 0.1 M HCl as the solvent due to its favorable solubility profile in acidic media. Levofloxacin is known to exhibit adequate stability under mildly acidic conditions, as it is less susceptible to hydrolytic degradation at low pH values than in neutral or alkaline environments. Within the scope of the present study, an extensive long-term stability study was not conducted. However, the short-term stability of both the stock and working standard solutions was evaluated over the analytical timeframe. The solutions were stored at room temperature and analyzed on the same day as preparation. No statistically significant changes in absorbance values were observed during the analysis period (RSD < 2%), confirming acceptable short-term stability under the specified conditions. In accordance with international method validation guidelines, solution stability was assessed to the extent necessary for the intended analytical application. The findings demonstrated that levofloxacin remained stable in 0.1 M HCl throughout the duration of the analytical procedure, and no additional stabilizing agents were required.

### 2.4. Standard Sample Preparation

We added 50 mg of levofloxacin and 30 mL of 0.1 M HCl to a 100 mL volumetric flask and then stirred. Then the solution was diluted with a solvent to obtain 0.5 mg/mL levofloxacin as the standard stock solution. 10 ml of the resulting solution is taken and placed in a 100 ml volumetric flask, and the volume is brought to the mark with the same solvent. The levofloxacin solution was prepared at 50 µg/mL.

The amount of prolonged levofloxacin in blood solution was calculated according to the following formula:

$$X=\frac{D_{pr}\times m_{st}\times V1}{D_{st}\times m_{pr}\times V2}\times 100$$

where *D_pr_* and *D_st_* are the optical density of the tested sample and the optical density of the sample of the standard, respectively. M_s_ and M_pr_ are the weight of the standard and sample in mg. In addition, 2 mL of plasma sample was placed in the test bottle and centrifuged at a speed of 4000 cycles/min for 5 min. Then 0.5 mL of the separated plasma was placed in a 50 mL volumetric flask with a volume, 20 mL of 0.1 M HCl was added to it, and it was mixed in a vortex mixer for 10 s. The volume of the solution was brought to 50 mL with the solvent.

### 2.5. Calibration Curve (Linearity, LOD and LOQ)

**Figure 1:**
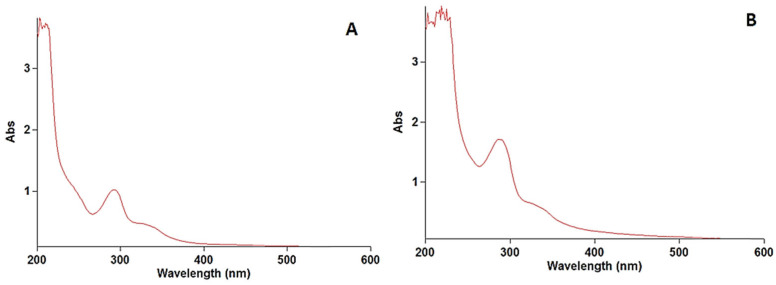
Spectra of levofloxacin standard (**A**) and prolonged levofloxacin (**B**) in blood.

The calibration curve was plotted by recording the UV–visible spectra of the prolonged levofloxacin at five concentrations (2.5, 5, 7.5, 10, 12.5 μg/mL) *vis* the intensity of the absorbance. Each concentration was tested in triplicate. Linearity was assessed by linear regression. A linear regression was constructed between the optical density (x) and the concentration (y) of the sample solution of the standard. Figure 1A demonstrates the obtained calibration plot in linear ranges from 2.5 to 12.5 μg/mL with an equation of y = 0.08696x + 0.3562 (*p*-value < 0.001, R^2^ 0.999). Figure 1B confirms the gradual increase in the absorbance intensity with increasing levofloxacin concentration. The determination of the limit of detection (LOD) and limit of quantitation (LOQ) was performed based on the standard deviations of y-intercept and the slope of the least square line parameters, as defined in the International Conference on Harmonization (ICH) guidelines. The calibration curve was constructed using five concentration levels, each analyzed in triplicate (n = 5). Thus, a total of fifteen determinations were performed for the establishment of the calibration model, as well as for the calculation of the LOD and LOQ values.

### 2.6. Accuracy and Recovery

Accuracy was evaluated at three concentration levels (80%, 100%, 120%) prepared in a biological matrix. Each level was analyzed in triplicate. Recovery (%) was calculated as the ratio of measured concentration to nominal concentration.

### 2.7. Precision

Precision was evaluated as intraday and interday repeatability. Six replicates of 5 μg/mL solution were analyzed. Precision is expressed as relative standard deviation (RSD%).

### 2.8. Specificity

Specificity was assessed by scanning blank matrix, diluent, and standard solutions over the range of 200–400 nm to evaluate potential interference.

### 2.9. Robustness Test

The robustness of the developed UV–Vis spectrophotometric method for levofloxacin quantification in rat plasma was evaluated by introducing small, deliberate variations in the experimental conditions. To assess the robustness of the proposed UV–Vis spectrophotometric method, planned minor variations were introduced, including:

- Analytical wavelength: ±2 nm around the selected λmax.
- pH of the plasma dilution buffer: ±0.2 units.
- Temperature: ±2 °C during sample preparation and measurement.
- Incubation time: ±5 min during plasma sample processing.

Minor variations in instrument gain and photometric parameters were observed. For each modification, absorbance measurements were performed in triplicate, and results were evaluated using %RSD (% relative standard deviation). A %RSD below 2% was considered acceptable to confirm method robustness.

### 2.10. Stability

The short-term stability of the stock and working solutions was evaluated under room-temperature conditions within the analytical timeframe.

### 2.11. Model Mixture and Matrix Preparation

Rats’ blood plasma samples were utilized as the matrix of the samples, which was mixed with the active substance in the ratio required during the determination of validation indicators.

### 2.12. Processing of the Obtained Results

Statistical analysis was performed to evaluate the relationships among the studied variables. Pearson’s correlation coefficient (r) was calculated to assess the strength and direction of linear associations. The analysis was conducted using Microsoft Excel 2013 (Microsoft Corp., Redmond, WA, USA) with the built-in CORREL function. Correlation coefficients were interpreted according to commonly accepted statistical criteria for weak, moderate, and strong associations.

### 2.13. Research Database Registration Number

This research work was classified under UDC 615.31, which encompasses the field of pharmacology and the chemistry of medicinal substances, and was systematized in accordance with its scientific scope and content. This conducted study underwent the required expert evaluation and registration procedures and was officially registered with the State Register of Scientific Research under SR No. 0111 4103. This registration confirms the scientific status of this research, its legal recognition, and the formal inclusion of its findings in the official scientific record.

## 3. Results

The Food and Drug Administration (FDA) recommends some fundamental parameters for the validation of each typical quantitative analytical method including the determination of the calibration curve, accuracy, precision, recovery, and selectivity of the proposed method with spiked samples. According to the set goal, a spectrophotometric method was developed for determining the amount of levofloxacin in the blood samples.

### 3.1. Solubility

Levofloxacin demonstrated satisfactory solubility in 0.1 M HCl, while limited solubility was observed in the other tested solvents. Therefore, an acidic medium was selected for further analysis.

Short-term stability testing showed no significant variation in absorbance values (RSD < 2%), confirming acceptable stability under analytical conditions.

### 3.2. Determination of Absorbance Maxima (λmax)

The stock solution of levofloxacin was scanned at wavelengths of 200 to 400 nm. The wavelength of the maximum absorbance of levofloxacin was found at 290 nm, which was used for preparing the calibration curve. The obtained results are illustrated in Figure 1.

### 3.3. Linearity, LOD and LOQ

**Figure 2:**
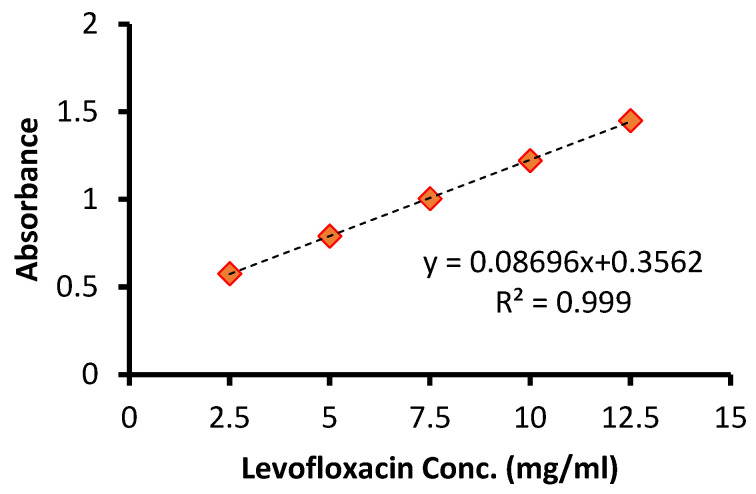
Calibration curve of levofloxacin determination.

A linear relationship between absorbance and concentration was observed within the range of 2.5–12.5 μg/mL. LOD and LOQ were calculated as 0.21 μg/mL and 0.62 μg/mL, respectively. Figure 2 demonstrates the obtained calibration plot in linear ranges. These values demonstrate improved sensitivity compared with several previously reported spectrophotometric methods (Table 1).

### 3.4. Accuracy and Recovery

The accuracy of an analytical method is calculated by dividing the calibration equation values and nominal concentration, which is defined as recovery percentage. Three concentrations of levofloxacin, including 2.5, 5, and 7.5 μg/mL, were prepared three time as low, medium, and high quantification concentrations. The intra- and interday recovery values ranged from 94.8 to 96.4% (Table 2). As shown in Table 3, the accuracy of the spectrophotometric quantification method in the model mixture containing levofloxacin and the indicators of the related statistical calculations met the relevant requirements.

### 3.5. Precision

The precision of an analytical method is expressed as the repeatability of the response by the relative standard deviation (RSD%). The FDA guidelines recommend evaluating method accuracy three times on the same and on different days of the experiments, which refer to intra- and interday precision, respectively. The levofloxacin solution (5 μg/mL) was prepared six times for the calculation of the RSD%. Table 4 and Table 5 indicate that the intra- and interday RSD values for levofloxacin determination were 0.2% and 0.25%.

### 3.6. Specificity

**Figure 3:**
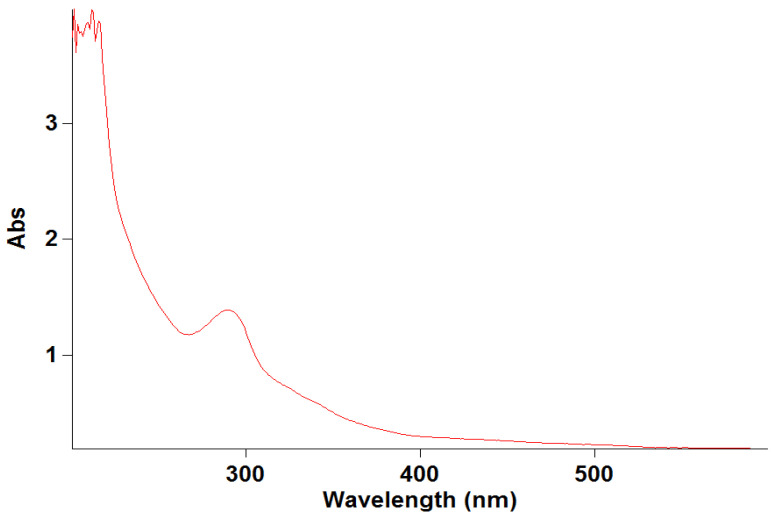
Specificity of levofloxacin.

The specificity of the developed method was determined by scanning the UV–visible spectra of diluted standard sample solutions of levofloxacin from 200 to 400 nm. The spectral homogeneity of the levofloxacin-spiked plasma samples was found to be similar to that of the standard solutions, confirming the absence of interference from the biological matrix. The specificity of the developed method was established to prove the absence of interference from diluent absorbance, which was part of the levofloxacin in the rat plasma. No interfering peaks were observed at 290 nm in the blank matrix samples, confirming method specificity (Figure 3). The obtained results are illustrated in Figure 3.

### 3.7. Robustness Test Results

The robustness of the developed spectrophotometric method was evaluated to determine its reliability under small, deliberate variations in experimental conditions, including wavelength, pH, and temperature. These variations were chosen to reflect minor deviations that may occur during routine analysis. The effect of these changes on the accuracy and precision of levofloxacin determination is summarized in Table 6.

### 3.8. Stability

The short-term stability of levofloxacin was evaluated under the conditions used in the present analytical procedure. Stock and working standard solutions prepared in 0.1 M HCl were stored at room temperature and analyzed on the same day as preparation. No statistically significant changes in absorbance values were observed during the analytical timeframe (RSD < 2%), indicating acceptable short-term stability. These findings confirmed that levofloxacin remained stable in 0.1 M HCl under the specified experimental conditions and that no significant degradation occurred during sample preparation and spectrophotometric measurement.

## 4. Discussion

### Comparative Advantages

The UV–Vis spectrophotometric method developed in this study offers several advantages over previously reported spectrophotometric methods and other analytical techniques for levofloxacin determination. Unlike earlier UV–Vis methods, which were primarily validated for pharmaceutical dosage forms such as tablets or ophthalmic solutions, the present method is specifically optimized for biological matrices, allowing reliable measurement of levofloxacin in rat plasma [14,15,16]. Compared with chromatographic techniques like HPLC or electrochemical methods, this approach is simpler, faster, and more cost-effective, requiring minimal sample preparation and no complex extraction procedures [17,18,19] Additionally, the method demonstrates high accuracy, precision, and selectivity even in the presence of a biopolymeric implant matrix containing chitosan and pectin, which may interfere with drug quantification. [20] This makes the proposed method particularly suitable for monitoring prolonged levofloxacin release from implantable drug delivery systems in preclinical studies, providing a practical and regulatory-compliant alternative to more complex and resource-intensive techniques.

Table 2 provides the brief overview of the figure-of-merits of some of the recent studies for levofloxacin determination in bio-fluids.

The present study describes the development and validation of a UV–Vis spectrophotometric method for the quantification of levofloxacin in rat plasma for the evaluation of a sustained-release antimicrobial mesh implant. While chromatographic techniques are generally considered the reference standards for fluoroquinolone determination in biological matrices, their application in routine preclinical screening may be limited by instrumental complexity, high operational costs, and time-consuming sample preparation procedures [21,22,23].

High-performance liquid chromatography provides superior selectivity and lower detection limits; however, it typically requires protein precipitation or solid-phase extraction, large volumes of organic solvents, and specialized analytical infrastructure. In contrast, spectrophotometric approaches are recognized for their simplicity and cost-effectiveness and have been widely applied for the analysis of levofloxacin in pharmaceutical dosage forms. Nevertheless, most previously reported UV–Vis methods were validated for tablets, ophthalmic preparations, or combined drug formulations rather than for biological matrices [24,25,26,27]. 

While chromatographic techniques such as high-performance liquid chromatography (HPLC) are widely used for the quantification of levofloxacin in biological matrices due to their high sensitivity and selectivity, planar chromatographic approaches including thin-layer chromatography (TLC) and high-performance thin-layer chromatography (HPTLC) have also been reported in the literature and provide additional context for analytical method selection for pharmaceutical analysis [28,29].

Several TLC-based methods have been developed for the separation and quantification of levofloxacin in pharmaceutical formulations. For example, a TLC method coupled with densitometric detection has been established for the simultaneous determination of levofloxacin hemihydrate and ambroxol hydrochloride in tablet dosage form, demonstrating good resolution and linearity, using silica gel 60 F254 plates and appropriate mobile phases. Additionally, HPTLC methods have been validated for simultaneous estimation of levofloxacin and other compounds such as ornidazole, providing simple, rapid, and accurate densitometric quantification with a linear response over the studied concentration range in solid dosage forms [28,29].

Although many TLC/HPTLC approaches were primarily developed for pharmaceutical tablets or combined formulations, their inclusion in the analytical discussion is valuable for a broader perspective. These planar chromatographic methods share several practical advantages—including simplicity, lower cost, minimal solvent use, and rapid analysis time—compared with traditional column chromatographic techniques. However, they also generally exhibit limited sensitivity and selectivity relative to spectrophotometric and HPLC-based methods when applied to complex biological matrices such as plasma, especially without additional sample preparation steps [28,29].

In contrast, the UV–Vis spectrophotometric method developed in the present study offers a practical alternative for routine bioanalysis of plasma without the need for complex extraction or chromatographic separation steps. It shows acceptable sensitivity (LOD of 0.21 µg/mL), precision, and accuracy with plasma samples, and its simplicity makes it especially suitable for preclinical pharmacokinetic studies involving sustained-release drug delivery systems. The present spectrophotometric approach therefore complements the existing TLC/HPTLC and chromatographic literature, particularly where resource constraints or operational simplicity are prioritized.

The analytical challenge associated with plasma determination lies in the presence of endogenous components capable of interfering with absorbance measurements. Therefore, unlike earlier studies focused on pharmaceutical forms, the present work specifically optimized sample preparation and wavelength selection to minimize matrix interference without introducing complex extraction steps. The absence of significant spectral overlap at the selected analytical wavelength confirms adequate selectivity under the proposed conditions. [30,31].

A further distinguishing feature of this study is its direct application to a composite implant system containing chitosan and pectin. Biopolymeric carriers may influence drug release kinetics and analytical recovery. The recovery and precision values obtained in this investigation demonstrate that the presence of polymer-derived components does not compromise quantification after plasma processing. This differentiates the present work from earlier spectrophotometric studies that did not address implant-associated matrices.

Validation was performed in accordance with internationally recognized regulatory recommendations ensuring compliance with accepted bioanalytical standards. The linearity coefficient approaching unity confirms a proportional detector response within the studied concentration range. The calculated limits of detection and quantification are sufficient for monitoring prolonged systemic concentrations expected from implant-mediated release. Although chromatographic techniques may achieve lower detection thresholds, the sensitivity observed here is appropriate for the intended pharmacokinetic application. 

The robustness study demonstrates that the proposed UV–Vis spectrophotometric method is stable against minor intentional variations. Wavelength, pH, and temperature changes did not significantly affect absorbance readings, with RSD values remaining below 2%. These findings indicate that the method is suitable for routine laboratory use and can be reliably applied under slightly different experimental conditions. Compared to other analytical approaches, the method is both simple and practically robust, making it particularly suitable for monitoring levofloxacin release from implantable drug delivery systems. [32].

Previous investigations have demonstrated that UV–Vis spectroscopy, while economical and accessible, may be less suitable for highly complex biodegradable composites if matrix interference is not adequately controlled. In the present study, optimization of plasma preparation conditions allowed reliable quantification without significant interference, thereby addressing one of the commonly reported limitations of spectrophotometric analysis in biological systems [33].

It is important to acknowledge that spectrophotometric detection does not provide the molecular specificity inherent to chromatographic separation. Consequently, the proposed method may not be appropriate for metabolite profiling or ultra-trace pharmacokinetic modeling. However, within the defined analytical objective—monitoring systemic levofloxacin concentrations during preclinical evaluation of a sustained-release mesh implant—the method demonstrates adequate accuracy, precision, and robustness [34,35].

In contrast to previously published reports primarily limited to pharmaceutical dosage forms, this study extends the applicability of UV–Vis spectrophotometry to plasma monitoring in the context of implantable drug delivery systems. The principal contribution of the present work lies in establishing a regulatory-compliant, analytically reliable, and operationally simplified method that balances performance with practical feasibility for routine preclinical assessment.

In contrast to all these studies, we will use, for the first time in the surgical field, a simple and rapid analytical method to determine the amount of levofloxacin in the sonicated blood of an elongating mesh implant impregnated with a mixture of chitosan, gelatin, tinctura propolis, citric acid, glycerin (base) and levofloxacin (active ingredient) prepared in vitro. The proposed method is simple, rapid, and sufficiently accurate. It is suitable for the determination of levofloxacin in blood samples and demonstrates its applicability for long-term monitoring. Details of sample preparation (Section 2.3, Section 2.4, Section 2.5, Section 2.6, Section 2.7, Section 2.8, Section 2.9 and Section 2.10), statistical analyses for accuracy and precision (Table 2, Table 4 and Table 5), and linearity assessment (R^2^, LOD, LOQ) are provided. The Discussion has also been expanded to compare our results with published methods. Furthermore, the method is appropriate for routine quality control analysis of levofloxacin in pharmaceutical dosage forms, particularly in mesh implant formulations. This increases the value of the analytical method set and makes it suitable for routine quality control analysis of levofloxacin in the future.

## 5. Conclusions

A UV–Vis spectrophotometric method was developed and validated for the quantification of sustained-release levofloxacin in rat plasma following administration via a hernia mesh. The method demonstrated satisfactory linearity over the concentration range of 2.5–12.5 μg/mL, with a limit of detection of 0.21 μg/mL, as well as acceptable accuracy, precision, robustness, and specificity. Owing to its simplicity, rapid analysis time, and cost-effectiveness, the proposed method represents a reliable analytical tool for the routine determination of levofloxacin in blood and for preclinical pharmacokinetic evaluation of implantable drug delivery systems.

## 6. Practical Application

The proposed method is of practical importance, as it can be applied in clinical and analytical laboratories for the monitoring of levofloxacin.

## Figures and Tables

**Table 1:** Parameters from calibration curve.

N	Levofloxacin Standard Concentration, µ/mL	Optic Density
1	2.5	0.577
2	5.0	0.791
3	7.5	1.004
4	10.0	1.221
5	12.5	1.449
Slope		0.0869
Y-intercept		0.3562
Determination coefficient (R^2^)		0.999
Linear function		y = 0.0869x + 0.3562

**Table 2:** Accuracy of the proposed method for levofloxacin determination.

Level of Recovery	Amount Bio. Matrix, mL	Amount of Std. Added, µg/mL	Total Amount Recovery, µg/mL	Recovery, %	Recovery Mean, %	SD	RSD, %	S.E.
80	1	2.5	2.41	96.40	96.27	0.611	0.635	0.35
80	1	2.5	2.42	96.80				
80	1	2.5	2.39	95.60				
100	1	5	4.74	94.80	94.67	0.115	0.122	0.07
100	1	5	4.73	94.60				
100	1	5	4.73	94.60				
120	1	7.5	7.18	95.73	95.60	0.231	0.242	0.13
120	1	7.5	7.18	95.73				
120	1	7.5	7.15	95.33				

**Table 3:** Figure of merits of some recent studies for levofloxacin determination.

Technique	Matrix	LOD (μg/mL)	Dynamic Range (μg/mL)	Ref.
Spectrophotometric	Plasma	0.63	2–20	[10]
Electrochemical	Serum	1.08 × 10^−6^	3.61 × 10^−6^	[11]
Electrochemical	Serum, urine, pharmaceutical	3.61	3.61–361	[12]
HPLC	Pharmaceutical	-	7.5–20.0	[13]
Spectrophotometric	Rat plasma	0.21	2.5 to 12.5	This research

**Table 4:** Inter- and intraday precisions of levofloxacin determination.

Nominal Concentrations (μg/mL)	Obtained Concentrations (μg/mL)	Recovery, %	Recovery Mean, %	Intraday Precision (SD)	Intraday Precision (S.E.)	Intraday Precision (RSD)
5	4.89	97.80	98.03	0.2	0.08	0.2
5	4.90	98.00				
5	4.91	98.20				
5	4.89	97.80				
5	4.91	98.20				
5	4.91	98.20				

**Table 5:** Interday precision of levofloxacin determination.

Nominal Concentrations (μg/mL)	Obtained Concentrations (μg/mL)	Recovery, %	Recovery Mean, %	Interday Precision (SD)	Interday Precision (S.E.)	Interday Precision (RSD)
5	4.74	94.80	94.87	0.24	0.09	0.25
5	4.73	94.60				
5	4.73	94.60				
5	4.75	95				
5	4.75	95				
5	4.76	95.20				

**Table 6:** Robustness Test Results for Levofloxacin Determination.

Parameter	Variation	Mean Absorbance	%RSD	Note
Wavelength	288 nm	0.742	1.12	Minor variation did not affect the method
Wavelength	292 nm	0.748	1.05	-
pH	6.9	0.745	0.98	-
pH	7.1	0.743	1.10	-
Temperature	23 °C	0.744	0.97	-
Temperature	27 °C	0.746	1.08	-

## Data Availability

The original contributions presented in this study are included in the article. Further inquiries can be directed to the corresponding author.
